# Towards Developing Bioresponsive, Self-Assembled Peptide Materials: Dynamic Morphology and Fractal Nature of Nanostructured Matrices

**DOI:** 10.3390/ma11091539

**Published:** 2018-08-27

**Authors:** Kyle M. Koss, Larry D. Unsworth

**Affiliations:** Department of Chemical and Materials Engineering, University of Alberta, 11487 89 ave, Edmonton, AB T6G 2M7, Canada

**Keywords:** self-assembling, peptides, RADA16, (RADA)_4_, MMP-2, nanoscaffold, Hausdorff dimension, fractal

## Abstract

(Arginine-alanine-aspartic acid-alanine)_4_ ((RADA)_4_) nanoscaffolds are excellent candidates for use as peptide delivery vehicles: they are relatively easy to synthesize with custom bio-functionality, and assemble in situ to allow a focal point of release. This enables (RADA)_4_ to be utilized in multiple release strategies by embedding a variety of bioactive molecules in an all-in-one “construct”. One novel strategy focuses on the local, on-demand release of peptides triggered via proteolysis of tethered peptide sequences. However, the spatial-temporal morphology of self-assembling nanoscaffolds may greatly influence the ability of enzymes to both diffuse into as well as actively cleave substrates. Fine structure and its impact on the overall effect on peptide release is poorly understood. In addition, fractal networks observed in nanoscaffolds are linked to the fractal nature of diffusion in these systems. Therefore, matrix morphology and fractal dimension of virgin (RADA)_4_ and mixtures of (RADA)_4_ and matrix metalloproteinase 2 (MMP-2) cleavable substrate modified (RADA)_4_ were characterized over time. Sites of high (glycine-proline-glutamine-glycine+isoleucine-alanine-serine-glutamine (GPQG+IASQ), CP1) and low (glycine-proline-glutamine-glycine+proline-alanine-glycine-glutamine (GPQG+PAGQ), CP2) cleavage activity were chosen. Fine structure was visualized using transmission electron microscopy. After 2 h of incubation, nanofiber networks showed an established fractal nature; however, nanofibers continued to bundle in all cases as incubation times increased. It was observed that despite extensive nanofiber bundling after 24 h of incubation time, the CP1 and CP2 nanoscaffolds were susceptible to MMP-2 cleavage. The properties of these engineered nanoscaffolds characterized herein illustrate that they are an excellent candidate as an enzymatically initiated peptide delivery platform.

## 1. Statement of Significance

The (RADA)_4_ peptide sequence boasts major benefits over other drug delivery systems. It is capable of forming a focal point of diffusion that houses and releases a variety of ligands in physiological conditions, and it is simple to add other functionally degradable peptide motifs during a one-step synthesis. As such, we added protease cleavable sites cued to injury to create a novel delivery system. However, the addition of peptides may inhibit the desired self-assembly of these nanoscaffolds. In our study we addressed this concern by observing nanoscale architecture and fractal features during self-assembly, which have been linked to diffusion in similar scaffolds. We also demonstrated that these materials can degrade with the hypothesized proteolytic cues.

## 2. Introduction

Peptides are the fastest growing segment of the pharmaceutical industry, and are generally considered the ideal therapeutic: specific, potent, small enough for diffusion, etc. [1]. Unfortunately, peptide therapeutics suffer from a major drawback; they are easily cleaved by naturally circulating proteases, and are therefore short lived. To circumvent this problem, peptide therapeutics have been incorporated into delivery vehicles with “on-demand” release cues, such as specific proteolytic cleavage sites [2,3]. Of the different systems, hydrogels that form nanofiber networks are promising in vivo carriers. Hydrogels offer dynamic and novel applications due to their unique properties, including: swelling with water, housing drugs and ligands, and being made into a multitude of mechanical and morphological configurations that provide multiple means of controlling release [4,5,6,7,8]. However, developing exact and tunable release mechanisms and morphological features can be extremely complicated, needing additional chemical synthesis involving toxic co-solvents, chemical triggers, or full transplantation to provide a fully formed network in vivo [9]. In addition, high throughput and systematic studies are imperative to drug discovery, requiring precisely timed drug release and cell response measurements, but are exceedingly complicated to perform on hydrogels [10,11]. With these limitations in mind, the ideal drug delivery hydrogel should incorporate accurate release cues, have reproducible gelation morphology, and be simple to synthesize. To these ends, controlled release peptide content can be precisely modulated and corresponding nanoscale morphology observed in a self-assembling peptides (SAPs).

Self-assembling peptides have been designed to spontaneously assemble into a nanoscaffolding material in the presence of aqueous salts, commonly found in vivo, by formation of non-covalent van der Waals forces, hydrogen bonds, and electrostatic interactions [12]. The resulting materials are capable of emulating pore and nanoscale fiber morphology of natural extra cellular matrices [13,14,15,16]. Further, these SAPs can be easily programmed by addition of peptide groups, including cleavage sites and drugs, during solid-phase synthesis, simplifying their overall fabrication into one predictable step. One such example, of a relatively well characterized SAP candidate for molecular-level programming, is the RADA16 or (RADA)_4_ construct (arginine-alanine-aspartate-alanine)_4_ [17,18,19,20]. This SAP peptide system has been used in tissue engineering and diffusion based release of drug and protein factors [16,20]. Furthermore, (RADA)_4_ has modified with a matrix metalloproteinase-2 (MMP-2) cleavable domain to study proteolytic degradation [21]. The morphology of the self-assembly process has been studied through various techniques, including circular dichroism, and atomic force, scanning, and transmission electron microscopy [22]. With the exception of short-term molecular modelling studies, comprehensive time-based studies for (RADA)_4_ assembly over a 24 h period have not been conducted [23]. Therefore, this study clarifies the matrix morphology as a function of assembly time, then attempts to interpret the impact of drug release as it relates to cellular activity.

Various forms of proteolytically triggered drug release systems have been studied, including nanoparticles and hydrogels, and have also been applied in therapy and imaging modalities [24]. In particular, the matrixin family is well known to be comprised of multiple enzymes, which are highly spatiotemporally regulated, within a large variety of diseases, including various forms of cancer and inflammatory responses [25]. Specifically, MMP-2 is abundantly secreted during injury and has been identified to cleave both high (glycine-proline-glutamine-glycine+isoleucine-alanine-serine-glutamine (GPQG+IASQ), CP1) and low (glycine-proline-glutamine-glycine+proline-alanine-glycine-glutamine (GPQG+PAGQ), CP2) affinity substrates, where the “+” indicates the sessile cleavage bond [26,27]. Previous work has incorporated these MMP-2 substrates into digestible polymer cross-linkers for the specific purpose of degrading dextran-based hydrogels to release embedded fluorophores [28]. That said, there are no known studies that have utilized these domains for the express purpose of understanding their effect on self-assembly of peptides and subsequent morphology.

Fractals are a measure of pattern symmetry that is present in every possible scale and appear in many facets of biology and life. In apparently disordered systems, fractal patterns may emerge and can indicate a relationship between the function of cells and biological materials, where no other information can be derived. In networked nanofibers, such as agarose and DNA-binding chromatin, fractal dimensions of morphology (i.e., porosity) are highly related to protein binding and diffusion coefficients [29,30]. Therefore, fractal nature may be strongly indicative of both the substrate-enzyme interaction and the diffusion within a matrix. One commonly used technique for classifying a fractal is the Hausdorff dimension analysis, which is an image-based technique employed to observe common patterns in otherwise random cell growth, laminin polymerization, nanotubes and nanovesicles, and other β-sheet forming SAPs [21,31,32,33,34,35]. Despite its wide use, no fractal network has been observed in (RADA)_4_, nor has it been linked to differences in growth or the addition of cleavage sites for drug release.

**Figure 1:**
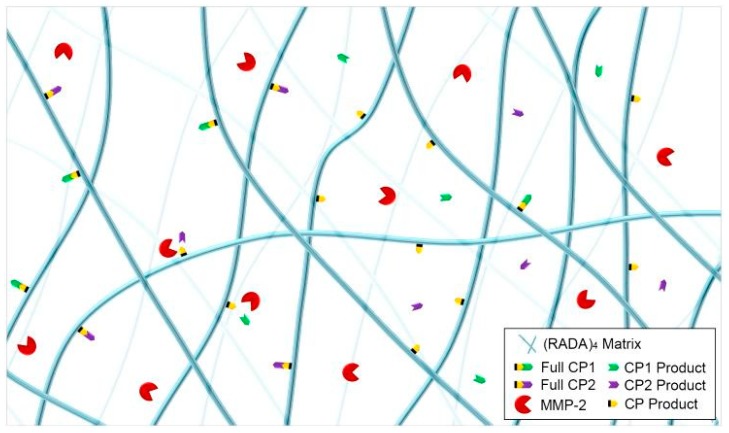
Schematic of nanofiber formation and MMP-2 cleavage of (RADA)_4_, (RADA)_4_-GG-GPQG+IASQ (CP1), and (RADA)_4_-GG-GPQG+PAGQ (CP2). “+” Denotes cleavage site and CP1, CP2, and CP products are IASQ, PAGQ, and (RADA)_4_-GG-GPQG, respectively.

In this study, self-assembly kinetics of (RADA)_4_ were assessed for systems that incorporated MMP-2 substrates (RADA)_4_-GG-GPQG+IASQ (CP1) and (RADA)_4_-GG-GPQG+PAGQ (CP2) to evaluate potential differences in resulting nanostructures, bulk bundling of nanofibers, fractal dimension, and cleavage (Figure 1). Morphology and assembly kinetics may vary with CP1 and CP2 and precise doping may be desired in future studies, therefore 100%, 75%, 50%, and 25% (RADA)_4_-CP1/CP2 were mixed with (RADA)_4_, and observed over 0, 1, 2, 4, 6, and 24 h. Resulting nanofibers were visualized using transmission electron microscopy (TEM) and corroborated by scanning electron microscopy (SEM). The resulting structures were statistically characterized by measuring bundle thickness and change in porosity. To estimate the fractal nature of these materials, which are related to protein binding and diffusion, the Hausdorff fractal dimensions were also estimated from these architectures, using box counting. MMP-2 was introduced to systems that exhibited extensive nanofiber bundling to evaluate if cleavage was still possible, where matrix-assisted laser desorption/ionization time of flight (MALDI-TOF/TOF) mass-spectrometry was used to characterize cleavage.

## 3. Results

### 3.1. Nanoscale Morphology

**Figure 2:**
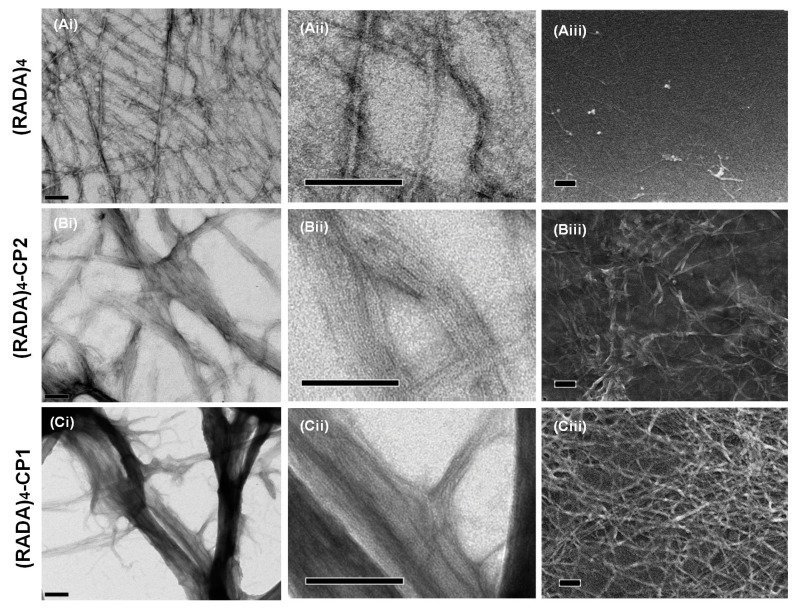
Electron microscopy of (**A**) (RADA)_4_, (**B**) (RADA)_4_-GG-GPQG+IASQ (CP1), and (**C**) (RADA)_4_-GG-GPQG+PAGQ (CP2). Low (**i**) and high (**ii**) magnification using transmission electron microscopy (TEM) are shown to demonstrate fibers present in bundles. Scanning electron microscopy (SEM) (**iii**) is also shown to corroborate TEM images. TEM samples were stained with 4% uranyl acetate and imaged at 0.5% *w*/*v* in TNC buffer upon 30 min of sonication and incubation at 37 °C for 24 h. SEM samples were sonicated similarly in TNC buffer, formed on glass coverslips at 37 °C, fixed with 2.5% gluteraldehyde, 4% paraformaldehyde in PBS (pH 7.4) for 24 h, then fixed again with 1% osmium tetraoxide in PBS for 1 h, and finally dehydrated and dried with ethanol and hexamethyldisilazane. Scale bars are all 100 nm.

Initially, 0.5% (*w*/*v*) peptide solutions of (RADA)_4_, (RADA)_4_-CP1, and (RADA)_4_-CP2 were allowed to assemble for 24 h in TNC buffer (Tris-NaCl buffer, 50 mM tris-HCl, pH 7.4, 150 mM NaCl, 1 mM CaCl_2_). The nanoscale morphology was then observed using TEM and SEM in order to determine if the ensuing forms were comparable using the different methods. Results demonstrate that the nanostructures along with their configurations were similar, not artifactual, and corroborated by the different techniques at low and high magnifications (Figure 2). Transmission electron microscopy was chosen as the tool to record experimental outcomes. The following section lists qualitative observations across groups.

**Figure 3:**
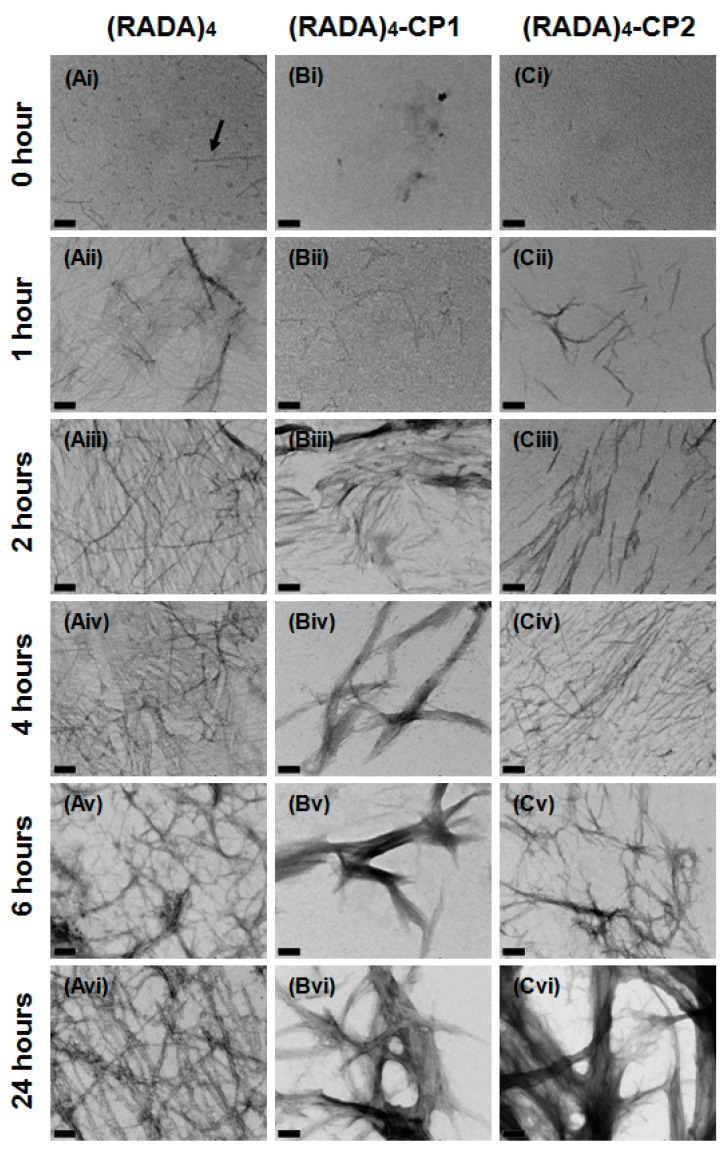
Transmission electron micrographs of (**A**) (RADA)_4_, (**B**) (RADA)_4_-GG-GPQG+IASQ, and (**C**) (RADA)_4_-GG-GPQG+PAGQ. Samples were sonicated for 30 min, incubated at 37 °C and pipetted onto grids at (**i**) 0, (**ii**) 1, (**iii**) 2, (**iv**) 4, (**v**) 6, and (**vi**) 24 h. All samples were stained with 4% uranyl acetate and imaged at 0.5% *w*/*v* in TNC buffer. Scale bars are 100 nm. Arrow (**Ai**) points to a discontinuous nanofiber.

**Figure 4:**
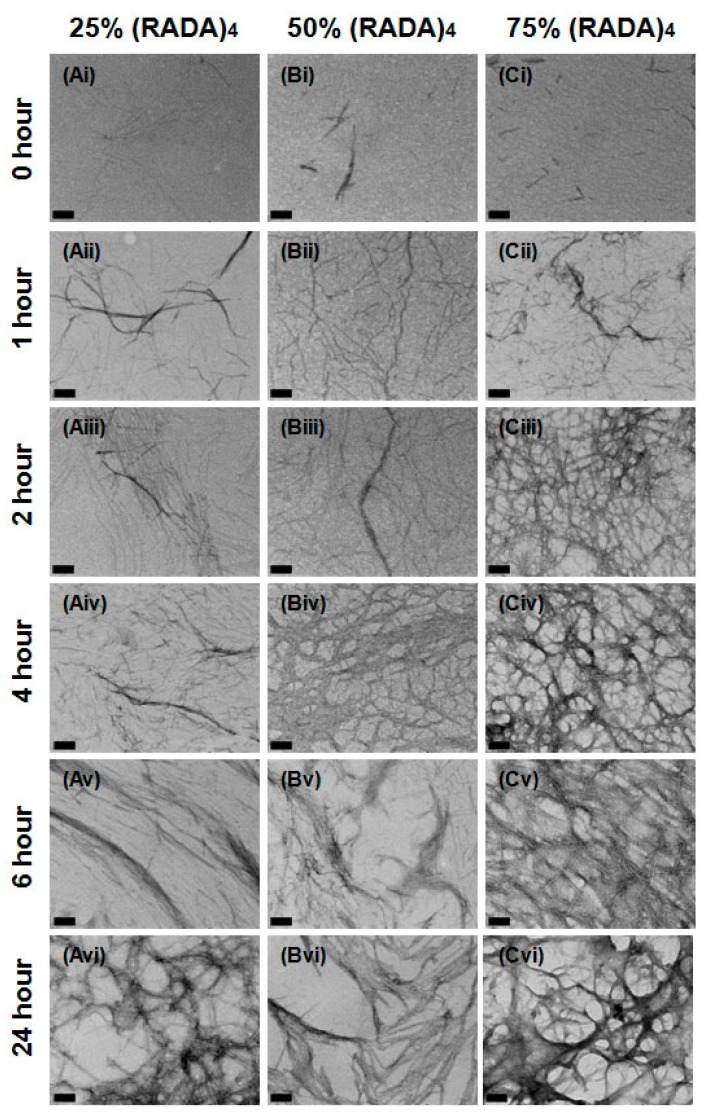
Transmission electron micrographs of (**A**) 25%/75%, (**B**) 50%/50%, and (**C**) 75%/25% (RADA)-GG-GPQG+IASQ/(RADA)_4_ at (**i**) 0, (**ii**) 1, (**iii**) 2, (**iv**) 4, (**v**) 6, (**vi**) 24 h. All samples were stained with 4% uranyl acetate and imaged at 0.5% *w*/*v* in TNC buffer upon 30 min of sonication and incubation at 37 °C. Scale bars are all 100 nm.

The progression of fiber assembly was followed at 0, 1, 2, 4, 6, and 24 h post-sonication (Figure 3). At 0 h, small 5–10 nm particles can be visualized in all images. The (RADA)_4_ and (RADA)_4_-CP2 trials already show individual nanofibers are present, indicating the rapidity with which fibers form. Nanofibers are present in all treatments past 1 h and networks continue to develop in complexity with time. Individual nanofiber thickness remains between 5–15 nm. After 1 h, individual threads have formed in (RADA)_4_, (RADA)_4_-CP1, and (RADA)_4_-CP2 and by 2 h, a loose patchwork of nanofibers exists in all groups. The network in (RADA)_4_ grows more defined, interconnected with time and evolved into a crosshatched arrangement of structures by 6 h. This formation articulates into thick bundled and branched fiber networks up to 150 nm, which were not observed for (RADA)_4_. These treatments transform into a spongelike, porous topography after 2 h for (RADA)_4_-CP1 and 6 h for (RADA)_4_-CP2. Strands were observed to form along this template with increasing density and intensity over the 24 h time line.

(RADA)_4_ cleavage peptides were mixed with (RADA)_4_ volumetrically to propose practical options for fine-tuned peptide cleavage and matrix re-structuring. Nanofiber emergence and structure was observed for 25%, 50%, and 75% volume per volume (*v*/*v*) (RADA)_4_ in (RADA)_4_-CP1, as they arise over 0, 1, 2, 4, 6, and 24 h post-sonication (Figure 4). Discontinuous fiber fragments, at least 15 nm in length, are apparent in 50% and 75% of (RADA)_4_ at time 0 h. At 1 h, a patchwork of individual threads, measuring 5–10 nm in diameter, can be recognized and are comparable for 25%, 50%, and 75% (RADA)_4_ in (RADA)_4_-CP1 mixtures. Nanofibers are present in all treatments past 1 h and these continue to increase in number and articulation over time. By 2 h, a loose web of fibers is present in most CP1 mixtures, which are similar in complexity to their pure unmixed forms (Figure 3 and Figure 4). The 75% (RADA)_4_, on the other hand, forms a different arrangement of threads at the 2 h time point; it displayed an intense filament bundling with a vesicular topography (Figure 4). This arrangement endures through to the 24 h end point, although the fiber networks and overall porosity become thicker and more defined. In the 25% mixture, after 4 h, the reticulum becomes more bundled but retains a crisscross filament arrangement. By 24 h, it bears the porous and curved architecture of its pure CP1 counterpart but is a nexus like pure (RADA)_4_. The 50% concentration loses its weave at 6 h, but does not effectively bundle and clusters in arrays of individual visible nanofibers. At 24 h, none of the diluted treatment nanofiber configurations were thickly bundled like CP1 alone, but are similar in overall topography (bundling and branching complexity) to pure (RADA)_4_ (Figure 3 and Figure 4).

**Figure 5:**
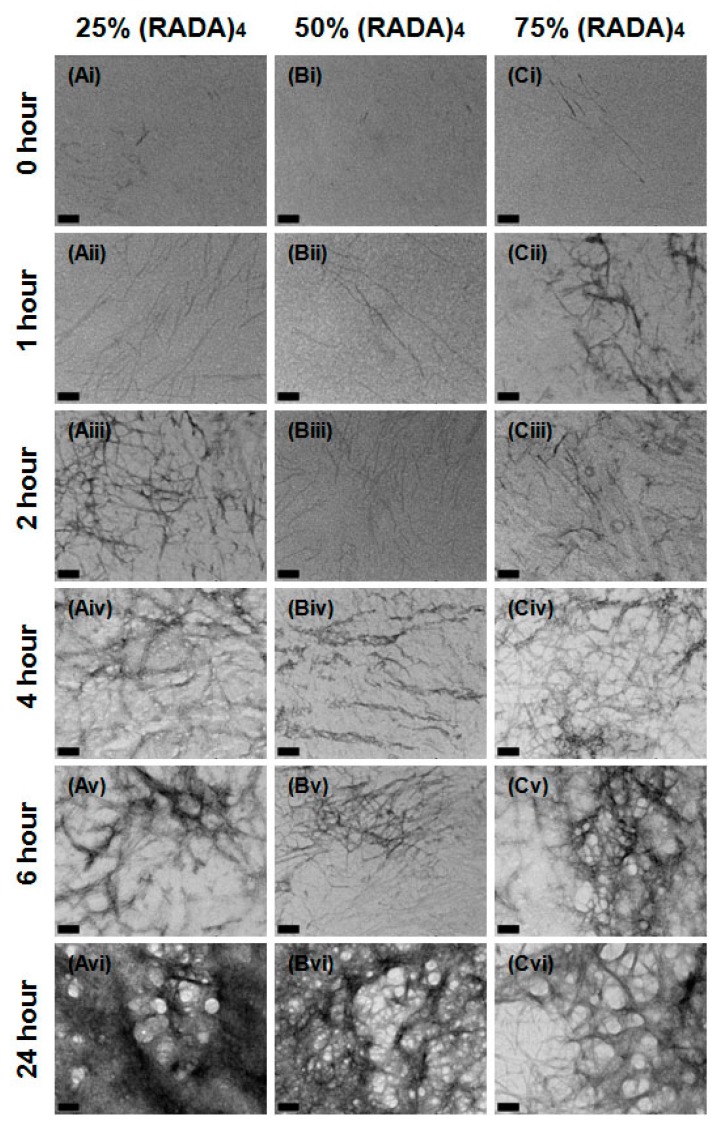
Transmission electron micrographs of (**A**) 25%/75%, (**B**) 50%/50%, and (**C**) 75%/25% (RADA)-GG-GPQG+PAGQ/(RADA)_4_ at (**i**) 0, (**ii**) 1, (**iii**) 2, (**iv**) 4, (**v**) 6, (**vi**) 24 h. All samples were stained with 4% uranyl acetate and imaged at 0.5% *w*/*v* in TNC buffer upon 30 min of sonication and incubation at 37 °C. Scale bars are all 100 nm.

Nanostructures resulting from (RADA)_4_-CP2 mixed to concentrations of 25%, 50%, and 75% (RADA)_4_, were observed at 0, 1, 2, 4, 6, and 24 h intervals after sonication (Figure 5). As in the (RADA)_4_-CP1 mixtures, nanofibers are visible within 1 h. The progression of thread formation and relation to one another follows a similar succession as in CP1 mixes. Nanofiber fragments emerged as single strands, measured approximately 15 nm in length were present in 25%, 50%, and 75% of (RADA)_4_ in (RADA)_4_-CP2. Between 1 until 6 h, a nanoscale latticework formed and was present in most CP2 mixtures. This webbed architecture was comparable in complexity to their pure peptide groups, which possess continuous nanofiber meshed matrices, with few diffuse fibers (Figure 3 and Figure 5). The exception was 75% (RADA)_4_ in (RADA)_4_-CP2 at 6 h, where nanofibers have formed a matt with a vesicular topography. This porous texture is similar to the fiber networks in 75% (RADA)_4_ in (RADA)_4_-CP1. Various vesicular architectures were observed in all treatments after 24 h of assemblage (Figure 5). Major differences are noteworthy at 24 h for (RADA)_4_ in (RADA)_4_-CP2 mixtures. In the 25% mix, a dense matt formed to overshadow the sample, and contained a few 50–100 nm pores. In the 50% CP2 mix, a less dense version of the fiber nexus was present and formed a greater number of 10–50 nm pores. The 75% concentration of (RADA)_4_ in (RADA)_4_-CP2, displayed a highly vesiculated architecture, with pores measuring from 100–150 nm in diameter, and were similar to those observed in 75% (RADA)_4_ mixed with (RADA)_4_-CP1 (Figure 4 and Figure 5).

### 3.2. Bundle Thickness and Fractal Dimension Analysis

**Figure 6:**
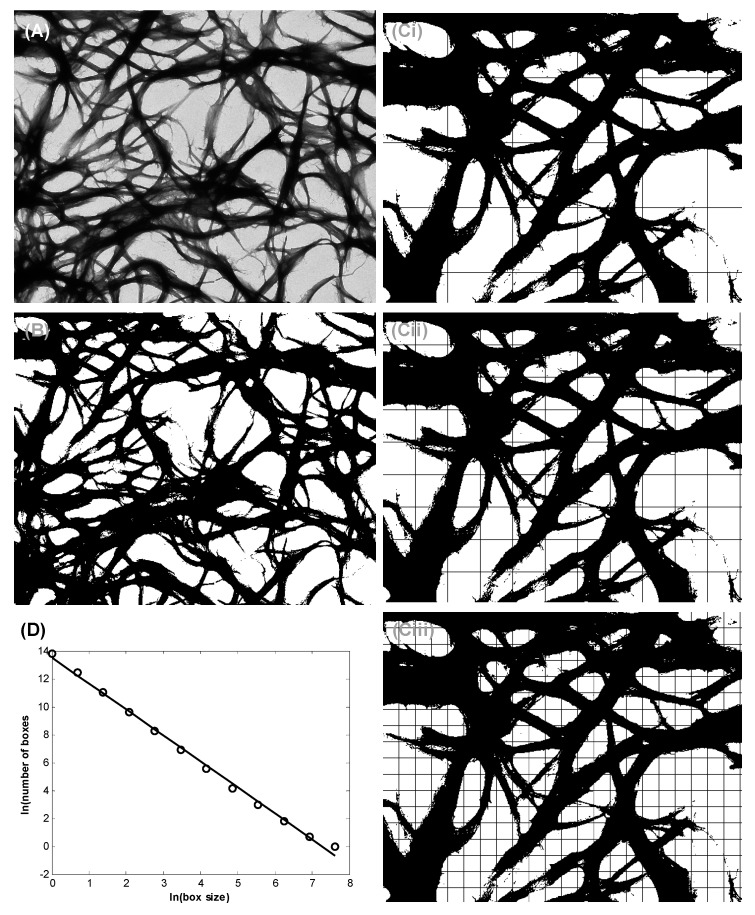
Example of image processing for Hausdorff boxcount method. (**A**) Transmission electron microscopy, (**B**) gray scale and background filtering off image. These include the Matlab functions for binary image conversion (im2bw) and background hole-filling (imfill). Also included are (**Ci**–**Ciii**) images with various grid sizes used to count pixels (*D*(*r*)) based on radius or dimension (*r*) for points of the box-count line, and (**D**) an example linear plot from the log of the box count curve. The negative slope of the linear curve is the box-count dimension (*D_H_*), this dimension being 1.8.

Bundle thicknesses of TEM images were quantified using a MATLAB box counting tool. Images were prepared for box counting by being placed on a grey scale and having their backgrounds subtracted. Pixels were counted base on their radius (*D*(*r*)), or grid size, and were plotted on a logarithmic scale. The slope of this line was considered the Hausdorff fractal dimension. An example is included in this section (Figure 6).

**Figure 7:**
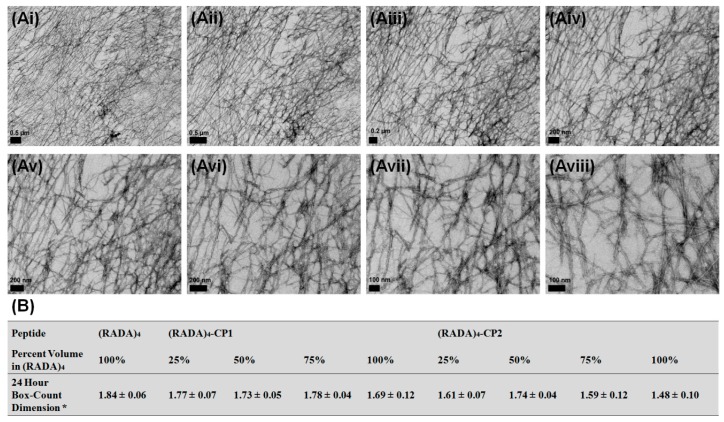
Transmission electron microscopy of (**Ai**–**viii**) various magnifications and (**B**) average Hausdorff box-count dimensions over 10 incremental changes in magnification (1400, 1800, 22,000, 28,000, 36,000, 44,000, 56,000, 71,000, 89,000, and 110,000× magnification). All samples were stained with 4% uranyl acetate and imaged at 0.5% w/v in TNC buffer upon 30 min of sonication and 24 h of incubation at 37 °C. Scale bars are all 100 nm, data analyzed represents mean ± SD over the magnifications and *n* = 3 experimental repeats.

As these fractals are a measure of continuous contour mapping present at any magnification, this was assessed with 10 magnifications (1400, 1800, 22,000, 28,000, 36,000, 44,000, 56,000, 71,000, 89,000, and 110,000), referenced from the scale. Averages and standard deviations for these were calculated (Figure 7). Continuous morphology appeared comparable by scale, and dimensions did not significantly change, no matter the magnification, nor were they below values expected in fractals. The previously discussed fractals bear significance at the observed scale.

**Figure 8:**
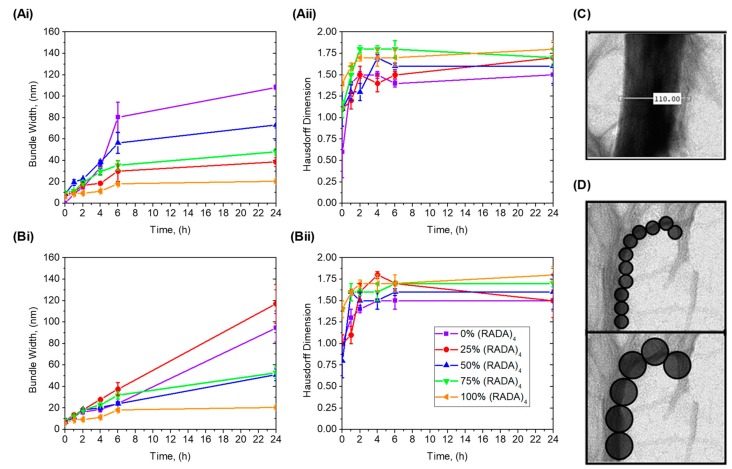
Bundle thickness and fractal dimension analysis for (RADA)_4_ systems. (**A**) Bundle thickness and (**B**) Hausdorff box-count dimensions of (RADA)_4_-GG-GPQG+IASQ (**i**) and (RADA)_4_-GG-GPQG+PAGQ (**ii**) mixtures with (RADA)_4_. Example images of (**C**) thickness measurement and (**D**) fractal contour tracing. Thickness image outlines the number of pixels across one bundle, which is used to calculate bundle width from scale bar. Mixtures include 100%, 75%, 50%, 25%, and 0% volume (RADA)_4_-GG-GPQG+IASQ/(RADA)_4_-GG-GPQG+PAGQ in (RADA)_4_. By sample, 50 random thicknesses were chosen for three independent images, from three independent experiments (i.e., *n* = 3, with 150 random measurements). Data analyzed represents mean ± SD, where line is to guide the eye only.

Fractal dimensions are summarized as a function of time for 0%, 25%, 50%, 75%, and 100% *v*/*v* (RADA)_4_ mixed with (RADA)_4_-CP1 and (RADA)_4_-CP2 (Figure 8Ai,Aii), Cross-sectional thicknesses of nanofibers and nanofiber bundling were measured and compared over the same time points and systems as the Hausdorff fractal dimension (Figure 8Bi,Bii). All bundle thicknesses increase with time, which is reflected in the TEM images. (RADA)_4_ does not bundle to too great an extent having nanofibers 15 ± 4 nm thick at 24 h. Twenty-five percent and 75% *v*/*v* (RADA)_4_ in (RADA)_4_-CP1 shared similar bundle thickness growth, 25% being 5–8 nm higher than 75% after 4 h and both approaching 50 ± 3 nm at 24 h. Fifty percent *v*/*v* (RADA)_4_ in (RADA)_4_-CP1 had comparatively higher bundle thicknesses than its 25% and 75% counterparts, having an average of 60 ± 8 nm at 24 h. Pure (RADA)_4_-CP1 bundle thicknesses were higher than these, being 100 ± 4 nm. (RADA)_4_-CP2 and all subsequent mixtures with (RADA)_4_ had similar bundle growth kinetics up until 6 h, and all within 20–40 nm thick by 6 h. By 24 h, 25% and 50% (RADA)_4_ in (RADA)_4_-CP2 also had similar bundle sizes both becoming 50 ± 5 nm. Pure (RADA)_4_-CP2, however, approached 100 ± 15 nm and 25% (RADA)_4_ in (RADA)_4_-CP2 bundles become as thick as 119 ± 18 nm, both being extensively thicker than other CP2 related mixtures. Each CP1 mixture rapidly reaches a plateau at 6 h, where a marginal increase of 10 nm occurred by 24 h. In contrast, the CP2 mixtures are continuously increasing in a slower and linear manner until 24 h, and had ultimately thicker bundling only when mixed with 25% (RADA)_4_. Fractal dimensions increase with time in every group. At 0 h, all experimental groups had lower range Hausdorff dimensions of approximately 1 or below except pure (RADA)_4_ and 75% (RADA)_4_ in (RADA)_4_-CP2. All systems reached a maximum dimension of 1.3–1.6 by 2 h. These dimensions were also observed across several magnifications (×14, ×18, ×22, ×28, ×36, ×44, ×56, ×71, ×89, ×110 K) and the variance in Hausdorff dimension were negligible (Figure 7).

### 3.3. MMP-2 Induced Peptide Cleavage

**Figure 9:**
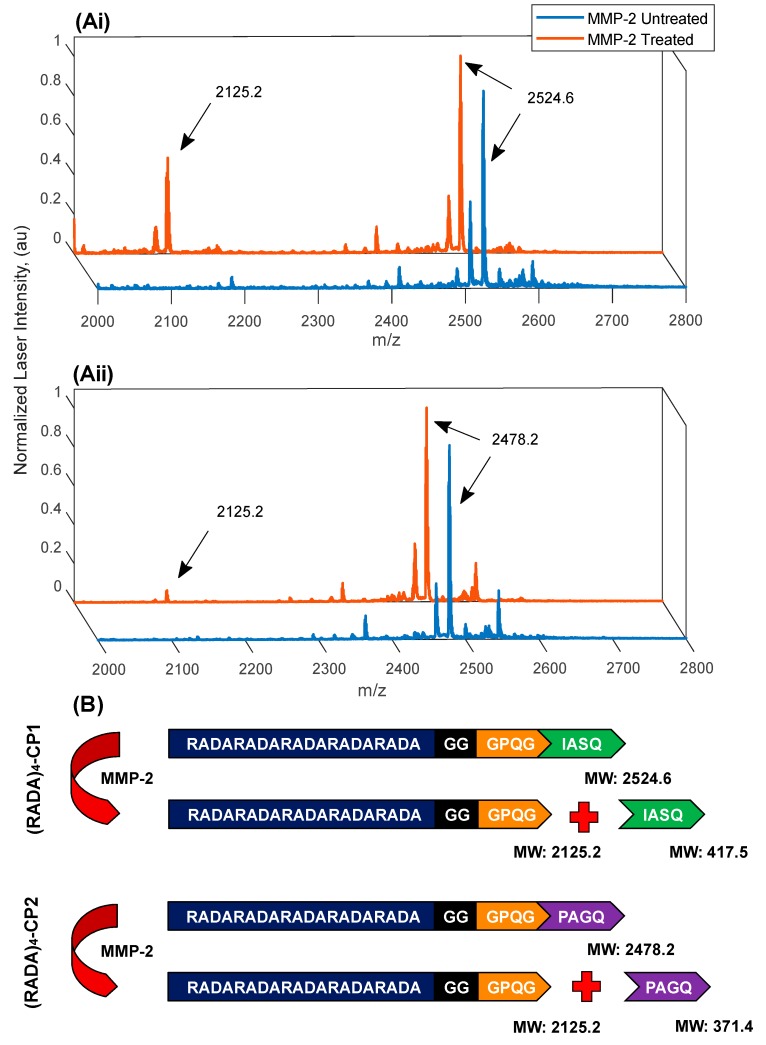
Matrix-assisted laser desorption/ionization time of flight (MALDI TOF/TOF) mass spectrometry of (**Ai**) 0.5% *w*/*v* (RADA)_4_-GG-GPQG+IASQ and (**Aii**) (RADA)_4_-GG-GPQG+PAGQ proteolysis. (**B**) Cleavage fragments are also shown. Incubated with 40 nM active MMP-2, in TNC buffer for three weeks at 37 °C. Substrate peaks are labelled 2524.6 *m*/*z* and 2478.2 *m*/*z*, representing theoretical molecular weights of (RADA)_4_-GG-GPQG+IASQ and (RADA)_4_-GG-GPQG+PAGQ, respectively. The peak 2125.2 *m*/*z* represents the molecular weight of the product for both substrates.

Enzymatic cleavage of high-activity (RADA)_4_-CP1 and low-activity (RADA)_4_-CP2 were observed using MALDI TOF/TOF mass spectrometry (Figure 9). These groups were chosen as the most bundled conditions, being the most proteolytically limited groups. The MMP-2 concentration of 40 nM was compared to the 0 nM control, in which no enzyme was present, over three weeks at 37 °C. Enzyme activity was quality controlled with zymography. Excessive enzyme and time was used to assure visible product formation in a diffusion limited system once all conditions of gelation have become static. Substrate peaks of 2525.2 *m*/*z* and 2478.8 *m*/*z* were visible in the enzyme and control groups for (RADA)_4_-P1 and (RADA)_4_-CP2, respectively. High and low product peaks were also present at 2125.1 *m*/*z* in the enzyme group for these respective groups. Although MALDI results are typically semi-quantitative, as peaks are influenced by both ionization potential and quantity, the peak for (RADA)_4_-CP1 was relatively higher than that of (RADA)_4_-CP2, showing that the sequence activity influences the product formation when present in nanoscaffold form. These values all matched the theoretical molecular weight of the substrates and products, showing that upon nanoscaffold formation substrate cleavage was possible.

## 4. Discussion

As long as the (RADA)_4_ peptide sequence was present, all peptides formed a nanofiber matrix. Small nanoparticles at 0 h suggest visible nucleation for (RADA)_4_ which may be due to more rapid self-assembly of (RADA)_4_ monomers. Individual nanofibers were formed by 1h in each condition indicating any initial self-assembly of nanofibers (not bundles) was finalized at this point. Both nucleation and rapid assembly were corroborated in initial studies by Zhang and Hauser [22]. Excessive bundle structures form with the addition of CP sequences (Figure 2, Figure 3, Figure 4 and Figure 5) and are likely a result of the amino acids added by CP1 and CP2 and their introduced intermolecular forces. GGGPQG, A and Q are shared between peptides. Glycines have no variable side chains and likely contribute minimal residual molecular interaction, therefore are often used as spacers in synthetic peptide combinations [36], as the double glycines (GG) were used for this purpose. Present in the both CPs, glycine may attenuate the bundling due to CP residues, but are not likely inhibiting the expected morphology due to (RADA)_4_. Glutamine may affect the electrostatic interaction associated with self-assembly, however hydrophobic interactions are considered dominant in (RADA)_4_ self-assembly [37]. The alanine residues are hydrophobic and may greatly contribute to the bundle formation. The presence of a cyclic pyrrolidine group integrated into the peptide backbone from proline may cause a cis-trans ‘bend’ in the peptide backbone and promote a bundle formation rather than the cross-linked mesh noted in pure (RADA)_4_ [38,39,40,41,42,43,44]. Small amounts of proline containing sequences have been shown to disrupt anti-parallel β-sheet formation, which occurs in (RADA)_4_ assembly, and may enhance the hydrophobic interactions that promote robust self-assembly. Comparing the two CPs, growth of the bundles is apparently faster in (RADA)_4_-CP1, but are ultimately thicker in (RADA)_4_-CP2. This quicker self-assembly may be due to the increased hydrophobicity added by the isoleucine residue in CP1, however another proline in CP2 potentially enhances the bundle thickness as further anti-parallel β-sheet disruption may be occurring. These systems vary in fiber bundling morphologies, suggesting their potential for proteolytic cleavage may change due to steric or diffusive hindrance, no longer reflecting their sequence specificity. Introducing pure (RADA)_4_ to these peptides in mixtures may allow a comparable morphology with higher surface area and ultimately assure cleavage.

Any addition of the pure (RADA)_4_ peptide sequence to the (RADA)_4_-CP1 and (RADA)_4_-CP2 enables self-assembly. Small nanofiber fragments are visible at 0 h for 50% and 75% *v*/*v* for (RADA)_4_-CP1 and all mixture of (RADA)_4_-CP2 with the addition of (RADA)_4_, but not the nanoparticles noted in (RADA)_4_. If nucleation does occur (noted previously as nanoparticles) it may be missed at the time points observed or is only visible in pure (RADA)_4_. Similar to pure (RADA)_4_ and (RADA)_4_-CP1, individual nanofibers are formed by 1 h in each condition suggesting any initial self-assembly, or nucleation, of individual nanofibers is finalized at this point. Overall, the nanofibers form in similar patterns when comparing (RADA)_4_ mixtures in (RADA)_4_-CP1 to it’s pure counterparts up until 2 h, except for a thicker porous matrix present in 75% (RADA)_4_ in (RADA)_4_-CP1. After this time point, the structures are comparable to (RADA)_4_ as no bundles are present in any time point. The thick porous matrix morphology persists in the 75% (RADA)_4_ group through to 24 h, which may represent an alternately shaped matrix for potential drug delivery than individual meshes or large bundles. Overall morphology is similar to between (RADA)_4_, (RADA)_4_-CP2 and its mixtures up until 6 h with 75% (RADA)_4_ in (RADA)_4_-CP2. This may be due to (RADA)_4_ having a dominant influence on the rate of self-assembly at 75%, and at 50%. However, at 25% this may be slower or have the same rate. The extensive bundling shown at 24 h for pure (RADA)_4_-CP2, may form thicker and more layered meshed networks when (RADA)_4_ is introduced to the mixture. This appears as a trend with the highest for 25% (RADA)_4_ in (RADA)_4_-CP2 and lowest for 75% (RADA)_4_ in (RADA)_4_-CP2, suggesting that the two peptides form alternate nanostructures that interact synergistically for form a composite when (RADA)_4_-CP2 is the larger part of the mixture. This was similarly noted in 75% (RADA)_4_ in (RADA)_4_-CP1, although this mixture was mostly (RADA)_4_. Self-assembly of (RADA)_4_-CP2 appears to be slower, forms thicker structures, and is better able to integrate with (RADA)_4_ than (RADA)_4_-CP1. The additional ‘bending’ of the second proline may allow (RADA)_4_ to favourably integrate into bigger structures over longer periods of time.

When quantified with bundle measurements, the (RADA)_4_ peptide sequence promoted self-assembly with added peptide groups. For (RADA)_4_-CP1 and its related mixtures, growth kinetics are higher in the pure CP1 group and thicker bundles are produced. The second to this is when 50% *v*/*v* (RADA)_4_ is present, not 25% *v*/*v*, which may be a result of counting the sparse clusters of nanofibers as bundles (Figure 4, at 6 and 24 h). 25% and 75% (RADA)_4_ in (RADA)_4_-CP1 are much closer to (RADA)_4_ in bundle thickness. Overall, this suggests that (RADA)_4_ plays a dominant role in self-assembly over (RADA)_4_-CP1, allowing for a matrix that retains similar bundle thickness and can load up to 75% drug bearing peptides. The bundle self-assembly was slower and more linear and allowed for thicker structures in pure CP2 and its mixtures when compared to CP1. Growth of pure (RADA)_4_-CP2 (Figure 3) was visibly linear within 24 h compared to (RADA)_4_-CP1 and this trend appeared in bundle thickness for mixtures (Figure 5) whenever these peptides were present. It is possible that the molecular bending from proline (once for CP1 and twice for CP2), which may enhance bundling, and the added hydrophobicity from isoleucine in CP1, which results in faster initial kinetics, are still present and progressively attenuated when (RADA)_4_ is introduced in mixture form.

With enough time, all meaningful fractal dimensions (above 1.3) were present in any mixture of (RADA)_4_, (RADA)_4_-CP1, and (RADA)_4_-CP2 and was comparable across multiple magnifications by 24 h. These values were equal or greater to that of a dendritic julia set, which is typically seen in neurite outgrowth [45]. Lomander et al. [34] derived a similar dimension of 1.34 for another β-sheet forming peptide SAP. This occurs by 2 h in every case, suggesting that growth kinetics of these shapes changes by this time point. Similarly, no significant bundling was visible in the TEM images nor was there statistically relevant bundling (*t*-test) derived from these images (Figure 8) for any mixture up to 2 h. This may be due to initial self-assembly requiring some nucleation to occur to generate full nanofibers from individual peptide monomers. Once full nanofibers are present, the fractal dimension reached a maximum, and growth was noted in further networking and bundling. Characteristic nanofiber growth is expected to be finished by this point [22]. Previous work by Fatin-Rouge and Bancaud have suggested that fractal properties in nanofiber networks may result in a system with a reduced apparent diffusion rate compared to non-fractal materials, whilst still allowing for protein-matrix interactions. This suggests that our system may perform as a proteolytic cued peptide delivery vehicle upon 2 h, which requires fractal dependent diffusive and protein-matrix (enzyme-substrate) interactions [29,30]. Even in the most highly structured bundles, MMP-2 cleavage is necessary to demonstrate this.

Adding the cleavage sequences of CP1 and CP2 to the (RADA)_4_ SAP allows for cleavage in the presence of high MMP-2 levels. However, their morphologies and growth kinetics vary extensively with increased bundling and lowered matrix density and networking. Cleavage rates and subsequent drug release may no longer be represented by their sequences and variable morphology related diffusion limitations, steric hindrances, and bulk release from bundles likely play a great role. Mixing these peptides allows for comparable nanostructures to pure (RADA)_4_ with the exception of 50% and 75% (RADA)_4_ in (RADA)_4_-CP2. As a result, this system can be modulated for exact drug loading in these morphologies, which can ultimately be tuned for on-demand delivery with MMP-2 endogenous to cancerous or inflammatory cells and tissues.

## 5. Materials and Methods

### 5.1. Materials

Methanol (99.8%), Ethanol (99.8%), dichoromethane (99.8%), acetonitrile (ACN) (99.8%), *N*-dimethylformamide (99.8%), piperidine (99.5% biotech. grade), hexamethyldisilazane (HMDS) (99%), osmium tetraoxide (4%), parafomraldehyde (36%), gluteraldehyde (8%), *N*-diisopropylethylamine (99.5% biotech. grade), 1-cyano-2-ethoxy-2-oxoethylidenaminooxy dimethylamino-morpholino-carbenium hexafluorophosphate (COMU) (97%), triisopropylsilane (99%), trifluoroacetic acid (TFA) (99%), α-cyano-4-hydroxycinnamic acid (HCCA) were acquired from Sigma (Oakville, ON, Canada) and used without further purification. Fmoc amino acids and wang resins were purchased from ChemPep (Wellington, FL, USA). Active human recombinant MMP-2 was acquired from EMD Millipore (Etobicoke, ON, Canada). Uranyl acetate and TEM grids were obtained from Ted Pella Inc. (Redding, CA, USA).

### 5.2. Peptide Synthesis

All self-assembling peptides, (RADA)_4_, (RADA)_4_-GG-GPQG+IASQ, and (RADA)_4_-GG-GPQG+PAGQ were synthesized using an ABI 433A Peptide Synthesizer. Fmoc chemistry was chosen and coupling was performed using 500 mM concentrations of COMU and ethyl (hydroxyimino) cyanoacetate. Fastmoc protocols were chosen and coupling was doubled and cycles were extended 15 min. All other protocols and methods were specified by the ABI 433A manual [46]. A cleavage cocktail of (96/2/2) TFA, water, and triisopropylsilane was used. ABI 4800 matrix-assisted laser desorption/ionization time of flight (MALDI-TOF/TOF) mass-spectrometry was used to assess sample masses. HCCA matrix concentrations of 10 mg/mL were suspended in 1:1 ACN:H_2_O (0.1% TFA). Peptides were purified to 95% or greater using high performance liquid chromatography (HPLC) and a Zorbax Eclipse C18 reverse-phase semi-preparative 9.4 × 250 mm column (Agilent Technologies, Santa Clara, CA, USA) using a H_2_O-ACN (0.1% TFA) loading. Purity was determined with HPLC with a Luna C18 reverse phase 4.6 × 250 mm column, by comparing the areas under the major curve to the minor curve areas in the HPLC spectra using Agitlent’s in-house software. MALDI mass spectra and chromatographs are presented in the supplemental section (Figure S1).

### 5.3. MMP-2 Enzymatic Treatment

Preparation for MMP-2 treatment was adapted from Chau et al. [21]. The gel was prepared by dissolving 1% weight per volume (*w*/*v*) of the peptides in TNC buffer (pH 7.4, 50 mM tris-HCl, 150 mM NaCl, 1 mM CaCl_2_). The amounts of (RADA)_4_-CP dissolved was normalized to so that the (RADA)_4_ sequence represented 1% *w*/*v* (peptide mass was multiplied by the molecular weight ratio of (RADA)_4_-CP over (RADA)_4_). These solutions were sonicated for 30 min and allowed to gel over 24 h. TNC buffer was carefully removed from the surface of the of the gel (10% of the volume) and refreshed, until pH of the removed buffer was balanced to 7.4. Active MMP-2 in TNC buffer was added as the final refreshed treatment 1:9 by volume for 40 nM enzyme concentration. TNC buffer, without MMP-2, was added as a control. These mixtures were incubated for three weeks at 37 °C to assure full digestion of the hydrogel. The samples were made soluble by diluting 1:1000 in 1:1 water/acetonitrile (0.1% TFA) and were subsequently sonicated for 30 min. The substrate and product fragments were measured by MALDI mass spectra, using the previously mentioned protocol.

### 5.4. Scanning Electron Microscopy

(RADA)_4_ samples were pipetted, in 20 µL aliquots, 12 mm round coverslips (Ted Pella, Redding, CA, USA) in 12-well plates. 80 µL of TNC buffer was gently added to this. All samples were collected after 30 min of sonication, and 24 h of incubation at 37 °C. The wells were topped up with 1 mL of fixative (2.5% gluteraldehyde and 4% paraformaldehyde in phosphate buffer saline (PBS), pH 7.4) and allowed to fix overnight for 24 h. Upon washing with 1 mL PBS, samples were fixed with 1% osmium tetraoxide for 1 h. Wells were washed with PBS, and dehydrated in 20% increments of ethanol with 30 min incubation until 100% ethanol. Ethanol was then replaced with HMDS in 25% increments with 30 min incubations. Samples were allowed to dry, and coated with carbon for 5 min. All SEM was performed on a JEOL JSM-6010LA InTouchScope scannning electron microscope (JEOL Ltd., Tokyo, Japan) with a 5 kV accelerating voltage.

### 5.5. Transmission Electron Microscopy

(RADA)_4_ samples were pipetted, in 5 µL aliquots, onto 200 mesh perforated formvar carbon coated copper grids (Ted Pella, Redding, CA, USA). Nanofiber emergence and structure was observed for 0%, 25%, 50%, 75%, and 100% volume per volume (*v*/*v*) (RADA)_4_ in (RADA)_4_-CP1 or (RADA)_4_-CP2. All samples were collected after 30 min of sonication, and 0, 1, 2, 4, 6, and 24 h of incubation at 37 °C. 5 µL of a 4% aqueous uranyl acetate stain was then applied to samples for 5 min. In between steps, sample and stain liquids were absorbed using filter paper wedges cut from Whatman filter paper. The negative staining technique was after Dawes [47] and Barroco, et al. [48]. All TEM was performed on a Philips FEI Morgagni transmission electron microscope (Hillsboro, OR, USA) at 80 kV accelerating voltage, and a tungsten thermionic emission source. Representative images were chosen and collected for each group.

### 5.6. Nanofiber Bundle and Hausdorff Dimension Analyses

All image processing and analysis was done using MATLAB^®^ R2012b (The Mathworks Inc. Natick, MA, USA). Bundles were measured by perpendicular distance across whole continuous nanofibers. These were chosen randomly 50 times for three images, repeated over three samples, for a total of 150 random measurements for each sample. The standard error mean was chosen across the three images.

Fractal dimensions were derived using a box counting method, similarly performed by Hochman–Mendez et al. [35] in laminin protein fibers. MATLAB code was modified from French and Costa and an example is shown in the appendices [48,49]. The Hausdorff dimension is an indication of likewise shapes in complex geometries based on specified points or members [50]. It is a method of quantifying fractals, but image-based calculation is not implied, therefore box counting was used to estimate these dimensions in this study. Using fixed grids with varying box sizes *r*, images were processed and the amount of boxes associated with a set *D*(*r*) were counted [51,52,53]. The following Equation (1) was then used to estimate the fractal dimension:

(1) $$D_{H}=-\frac{\Delta \left[\log D\left(r\right)\right]}{\Delta \log\left(1/r\right)}$$

where *D_H_* is the Hausdorff–Besicovitch dimension or simply the box count fractal dimension. All images were processed as full images in greyscale, and the functions im2bw and imfill were used to convert to binary and fill regions, respectively. An example of this and the resulting box count curve is shown in the results (Figure 6). All samples were analyzed based on the mean and standard deviation of *n* = 3, where significance was based on *p* < 0.05 (*t*-test) for individual discussed values.

## 6. Conclusions

The temporal growth morphology and fractal dimension for a (RADA)_4_ hydrogel were studied as a function of C-terminal tethered MMP-2 substrate (GPQG+IASQ (CP1), GPQG+PAGQ (CP2)), and overall substrate concentration within the matrix. This was done for the express purpose of elucidating the effect these parameters have upon matrix morphology with assembly time, and the ability of the system to be enzymatically cleaved; all of which is crucial to the development of MMP-2 induced release of peptides from these SAP systems. Both substrate modified (RADA)_4_ peptides self-assembled into nanofibers and resulted in a significant bundled morphology compared to (RADA)_4_. To maintain a similar morphology to (RADA)_4_, (RADA)_4_-CP1/CP2 were doped with 25%, 50%, and 75% *v*/*v* pure (RADA)_4_. Any amount of (RADA)_4_ added to CP1 mixtures demonstrated consistent architecture to its unmodified parent peptide, however the CP2 formula was only comparable to (RADA)_4_ with 75% addition. A densely meshed architecture was noted with 50% and 25% (RADA)_4_ in (RADA)_4_-CP2, potentially allowing for different release kinetics. Fractal dimension reached a meaningful plateau at 2 h, however, nanofiber bundling continued after this time in all cases. The systems that exhibited the most bundling, (RADA)_4_-CP1/CP2, were still susceptible to MMP-2 cleavage. As a potential drug delivery system, these peptide mixtures are capable of predictable morphologies for in situ nanoscaffold formation, and tunable cleavage substrate addition for on-demand release. Undergoing enzyme kinetic and cell response studies, this system may demonstrate tremendous merit in tissue engineering applications and as a therapeutic for several inflammation and cancer related illnesses.

## Supplementary Materials

The following are available online at http://www.mdpi.com/1996-1944/11/9/1539/s1. Figure S1: Representative (i) HPLC (i) and (ii) MALDI TOF/TOF mass spectrometry (ii) of purified peptide (RADA)_4_, (RADA)_4_-GG-GPQG+IASQ, and (RADA)_4_-GG-GPQG+PAGQ, shown in A, B, and C, respectively. Purities were determined to be above 95% by measuring the comparative areas under the major curve in the HPLC spectra. Major peaks in the MALDI spectra are at 1671.8, 2525.2, and 2478.8 m/z which reflect the theoretical molecular weights. All peaks were normalized to their respective maxima.
