# Supplementary material for: Towards Developing Bioresponsive, Self-Assembled Peptide Materials: Dynamic Morphology and Fractal Nature of Nanostructured Matrices
Source: Materials (Basel). 2018 Aug 27;11(9):1539. doi: 10.3390/ma11091539 (PMC6164152; doi:10.3390/ma11091539)
Supplement: Supplementary file 1 [file materials-11-01539-s001.pdf]

Towards Developing Bioresponsive, Self-Assembled Peptide Materials: Dynamic Morphology and Fractal Nature of Nanostructured Matrices

Kyle Koss and Larry Unsworth

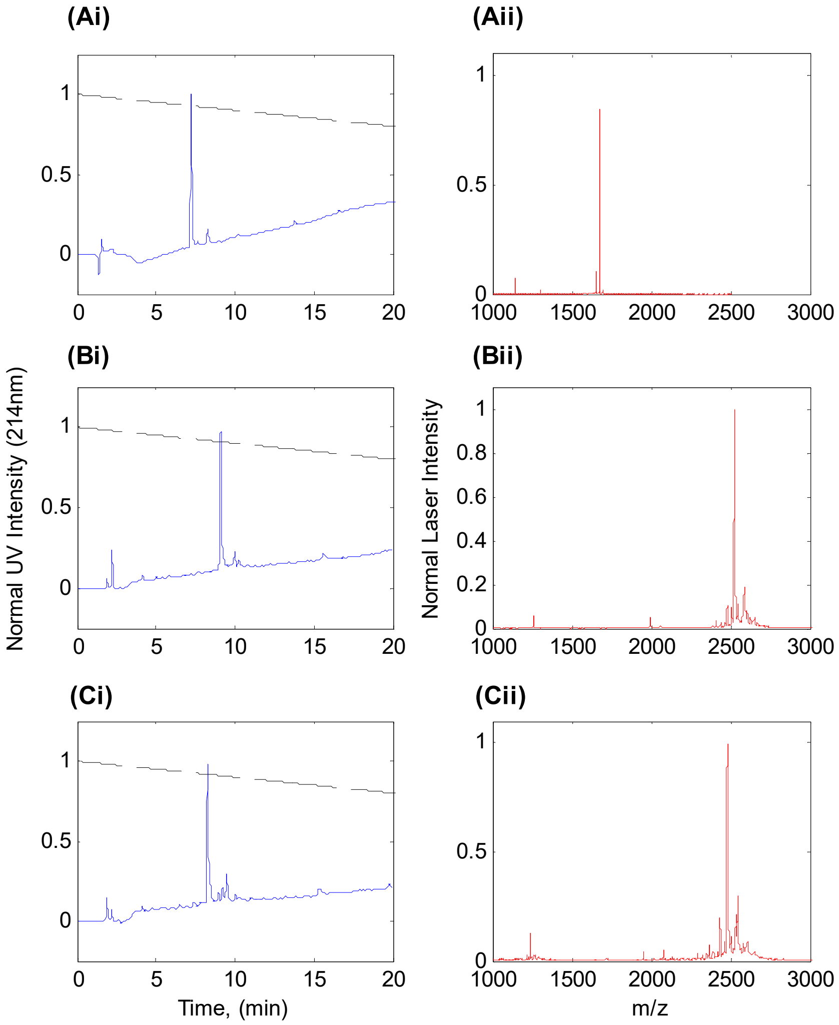

Figure S1. Representative **(i)** HPLC (i) and (ii) MALDI TOF/TOF mass spectrometry (ii) of purified peptide $(RADA)_4$, $(RADA)_4$-GG-GPQG+IASQ, and $(RADA)_4$-GG-GPQG+PAGQ, shown in A, B, and C, respectively. Purities were determined to be above 95% by measuring the comparative areas under the major curve in the HPLC spectra. Major peaks in the MALDI spectra are at 1671.8, 2525.2, and 2478.8 m/z which reflect the theoretical molecular weights. All peaks were normalized to their respective maxima.
